# Supplementary material for: Excessive excision of correct nucleotides during DNA synthesis explained by replication hurdles
Source: EMBO J. 2020 Feb 9;39(6):e103367. doi: 10.15252/embj.2019103367 (PMC7073461; doi:10.15252/embj.2019103367)

Table of contents:

1. Legends to the Appendix Figures S1-S3
2. Appendix Figures S1-S3

Legends to Appendix Figures:

Appendix Figure S1. A. Moles of nucleotides incorporated (i) and excised (ii) per mole of substrate DNA per second during leading strand synthesis reactions performed with constant 500 µM dTTP and indicated dVTP concentrations.

Bars show data from two individual experiments.

B. Polymerase and exonuclease activities during leading strand synthesis measured with α-^{32}P-dATP. i. Experimental design. 500 µM dTTP and 150 µM dVTPs were used for these experiments. ii. Representative TLC. iii-v. Moles of nucleotides incorporated (iii) and excised (iv) per mole of substrate DNA during leading strand synthesis and Pol/Exo ratios (v) for reactions performed with α-^{32}P-dATP and similar reactions carried out with α-^{32}P-dGTP (reproduced from Figure 1F, uncoupled leading strand synthesis for comparison).

Circles in two shades of the same color show data points from two individual experiments. Lines show the linear fits of the mean data. Bars show data from two individual experiments.

Appendix Figure S2. Effect of gp2.5 concentration on Pol- and Exo-activities. A. Schematic of the strategy used for the experiments. B and C. Pol-activity (B) and Exo-activity (C) expressed as moles of nucleotides incorporated and excised per mole of substrate DNA. D. Pol/Exo ratios as determined with 0 µM, 8 µM and 16 µM T7 gp2.5. Pol/Exo data are repeated here from Figure 5H.

Bars show data from two individual experiments.

Appendix Figure S3. Exonuclease activity increases when helicase is slowed. A. Experimental set-up used to determine fractions of dGMP incorporated and dGMP excised during leading strand synthesis reactions performed with 10 µM dVTPs and increasing concentration of dTTP. B-C. Polymerase activity (B) and exonuclease activity (C) in reactions performed with increasing dTTP concentrations (expressed as moles of nucleotides incorporated or excised per mole of substrate DNA). (D) Moles of dGDP produced per mole of substrate DNA.

Circles in two shades of the same color show data points from two individual experiments.
Lines show the linear fits of the mean data.

Appendix Figure S1

A. Pol- and Exo-activities measured during leading strand synthesis

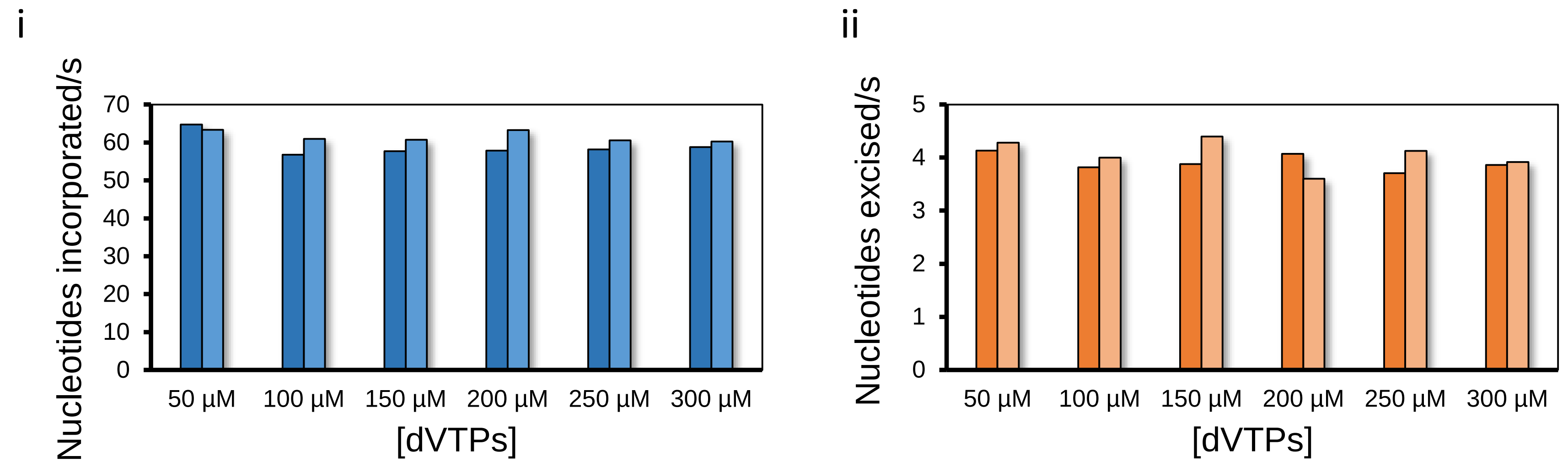

B. Pol/Exo ratio measured during leading strand synthesis with α-^{32}P-dATP

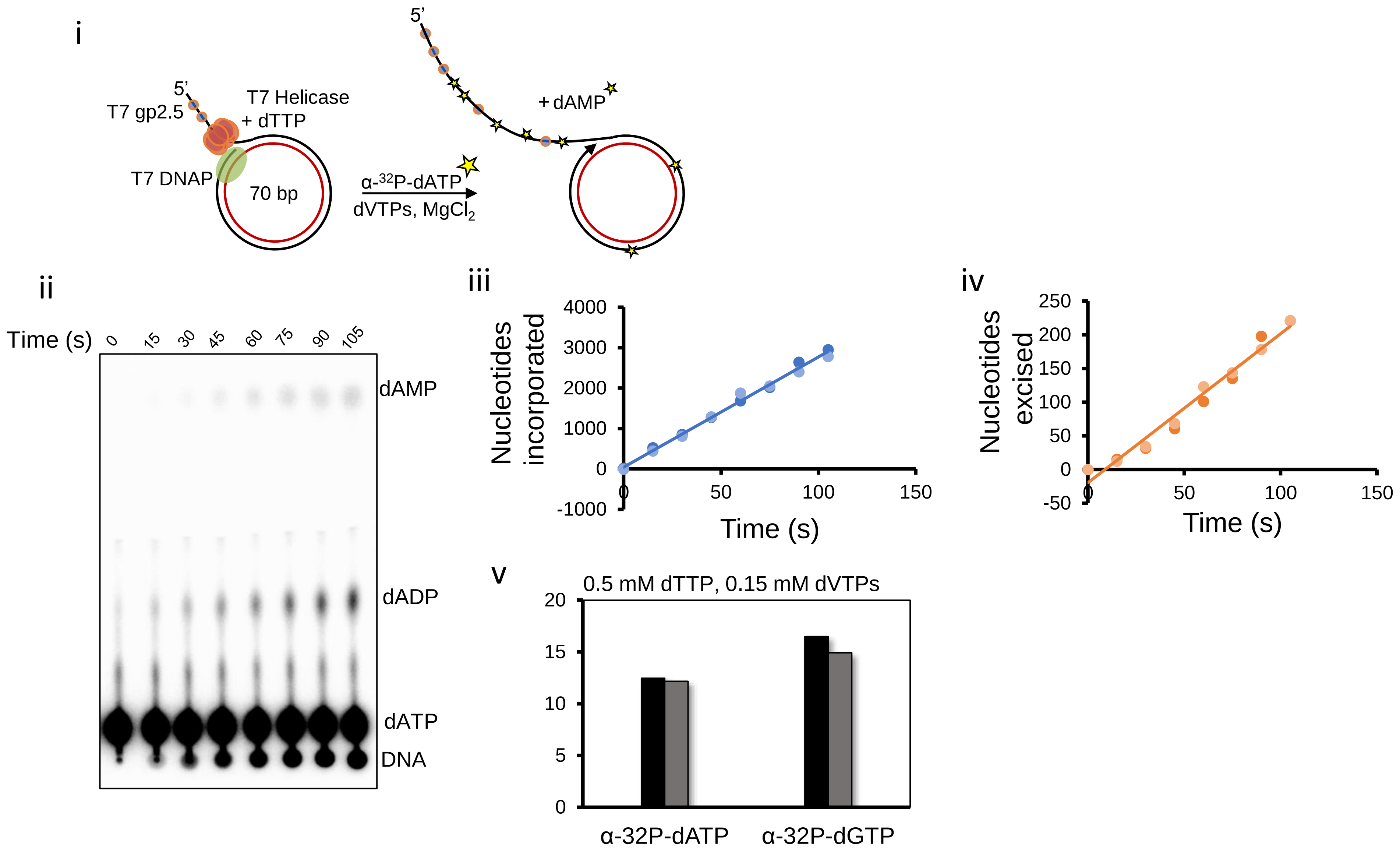

Appendix Figure S2

Effect of T7 gp2.5 concentration on Pol- and Exo-activities

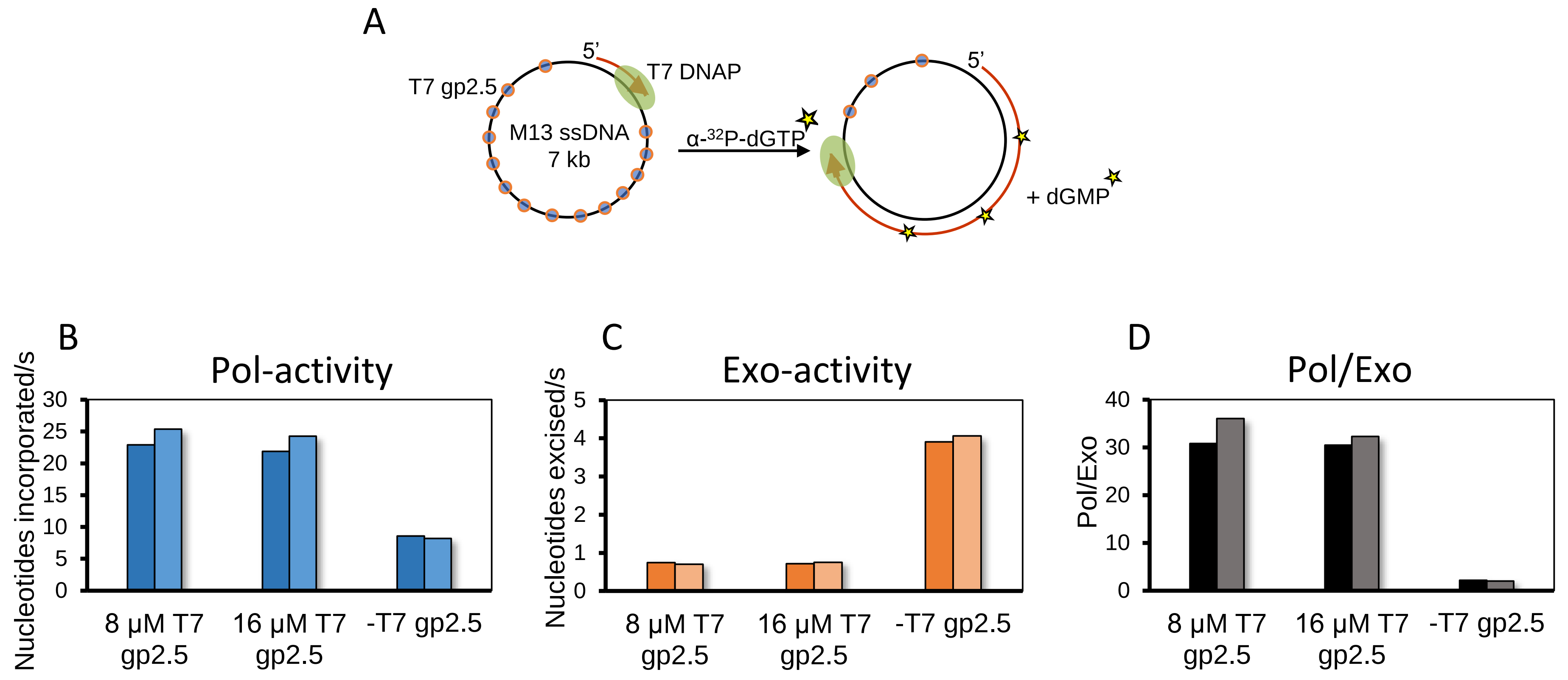

Appendix Figure S3

Effect of dTTP concentration on Pol- and Exo-activities

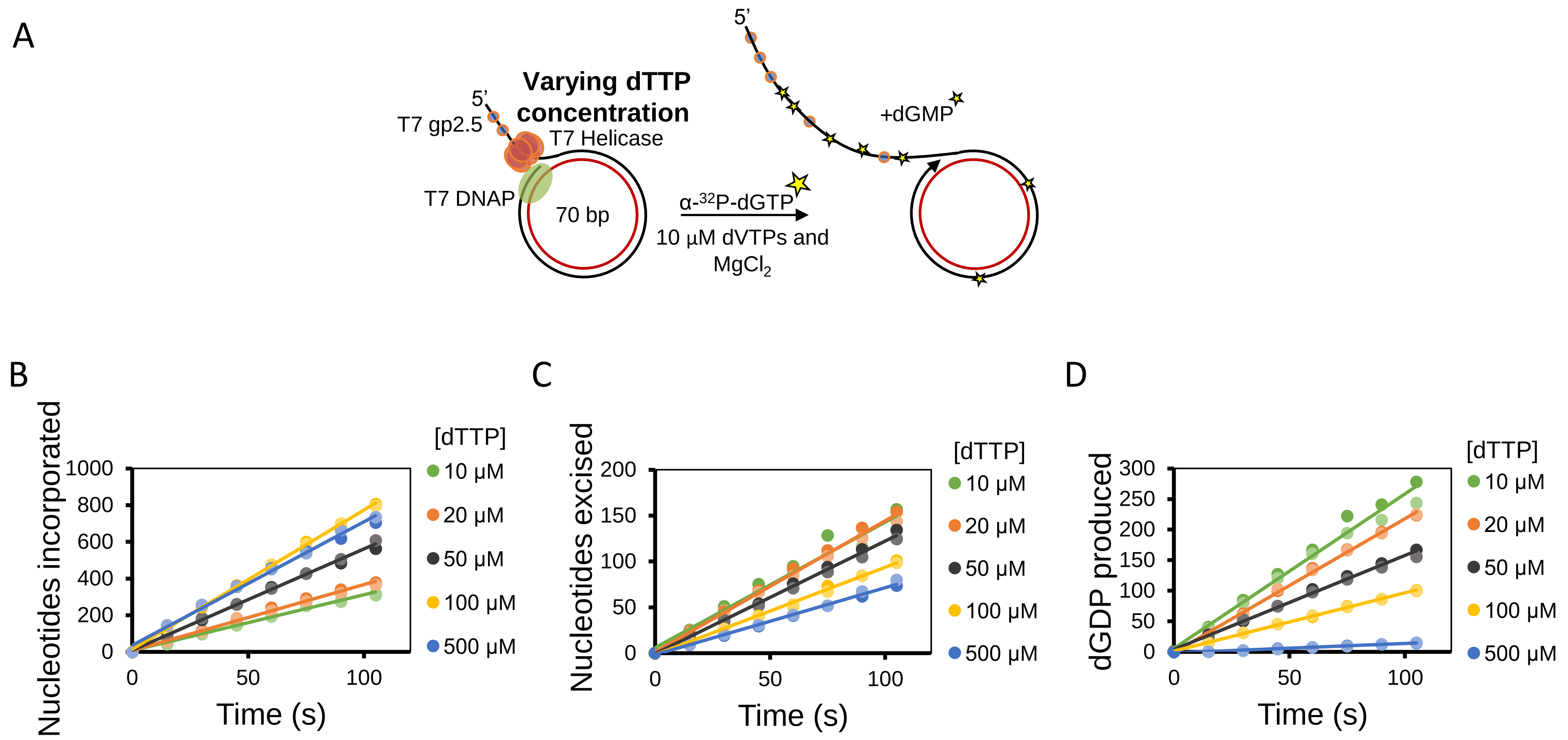

Supplement: Supplementary file 1 — Appendix [file EMBJ-39-e103367-s001.pdf]
