# Supplementary material for: Excessive excision of correct nucleotides during DNA synthesis explained by replication hurdles
Source: EMBO J. 2020 Feb 9;39(6):e103367. doi: 10.15252/embj.2019103367 (PMC7073461; doi:10.15252/embj.2019103367)
Supplement: Supplementary file 2 — Expanded View Figures PDF [file EMBJ-39-e103367-s002.pdf]

Expanded View Figures

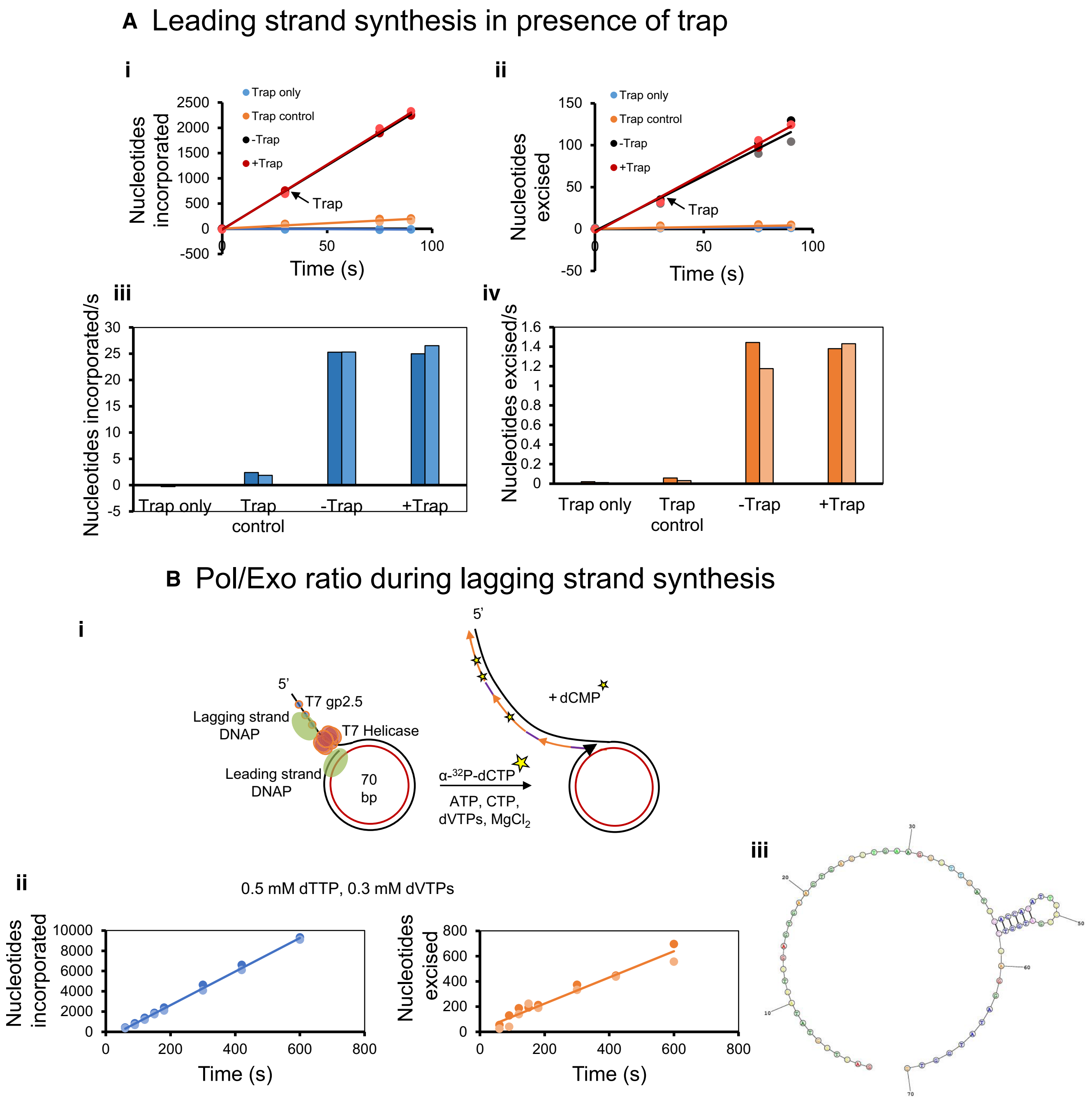

Figure EV1. Polymerase and exonuclease activities measured during ongoing leading and lagging strand syntheses.

A Leading strand synthesis in the presence of 800 nM protein trap added at 30 s after reaction initiation. Moles of nucleotides incorporated (i and iii) and excised (ii and iv) per mole of replisome during leading strand synthesis with minicircle DNA substrate. “Trap only” did not have minicircle DNA substrate, “Trap control” reactions were performed with protein trap mixed with minicircle DNA before the addition of replication proteins, and no trap was added in “-Trap” reactions.

B Pol/Exo ratio during lagging strand synthesis. Lagging strand reactions were performed with minicircle DNA substrates in the presence of ATP and CTP and α-^{32}P-dCTP. (i) Time courses of nucleotides incorporated and excised (ii) (in moles/mol of DNA) during lagging strand synthesis. (iii) Secondary structure in the lagging strand template. Prediction was made using RNAstructure (Xu & Mathews, 2016).

Data information: Circles in two shades of the same color show data from two individual experiments. Lines show the linear fits of the mean data. Bars show the data from two individual experiments.

 © 2020 The Authors

Figure EV2. Excessive exonuclease activity is not due to contaminating exonucleases.

A Leading strand synthesis in the presence of T7 DNAP exo−. (i) Experimental design. (ii) TLC spots representing fractions of dGMP excised (left panel) and incorporated (right panel) during leading strand synthesis reactions conducted with T7 DNAP exo+ and T7 DNAP exo−. (iii) Time courses of nucleotides excised (left panel) and incorporated (right panel) (in moles/mol of DNA) during leading strand synthesis reactions conducted with T7 DNAP exo+ and exo−.

B Control experiments on primed M13 mp18 ssDNA substrate. (i) Experimental design and reactions spotted on TLC. (ii) Resolved spots on TLC showing fractions of dGMP excised (top) and incorporated (bottom). (iii) Fractions of dGMP incorporated and excised as quantified from spot intensities in (ii).

Data information: Circles in lighter shades show data from two individual experiments. Circles in darker shades of the same color represent the mean data.

© 2020 The Authors

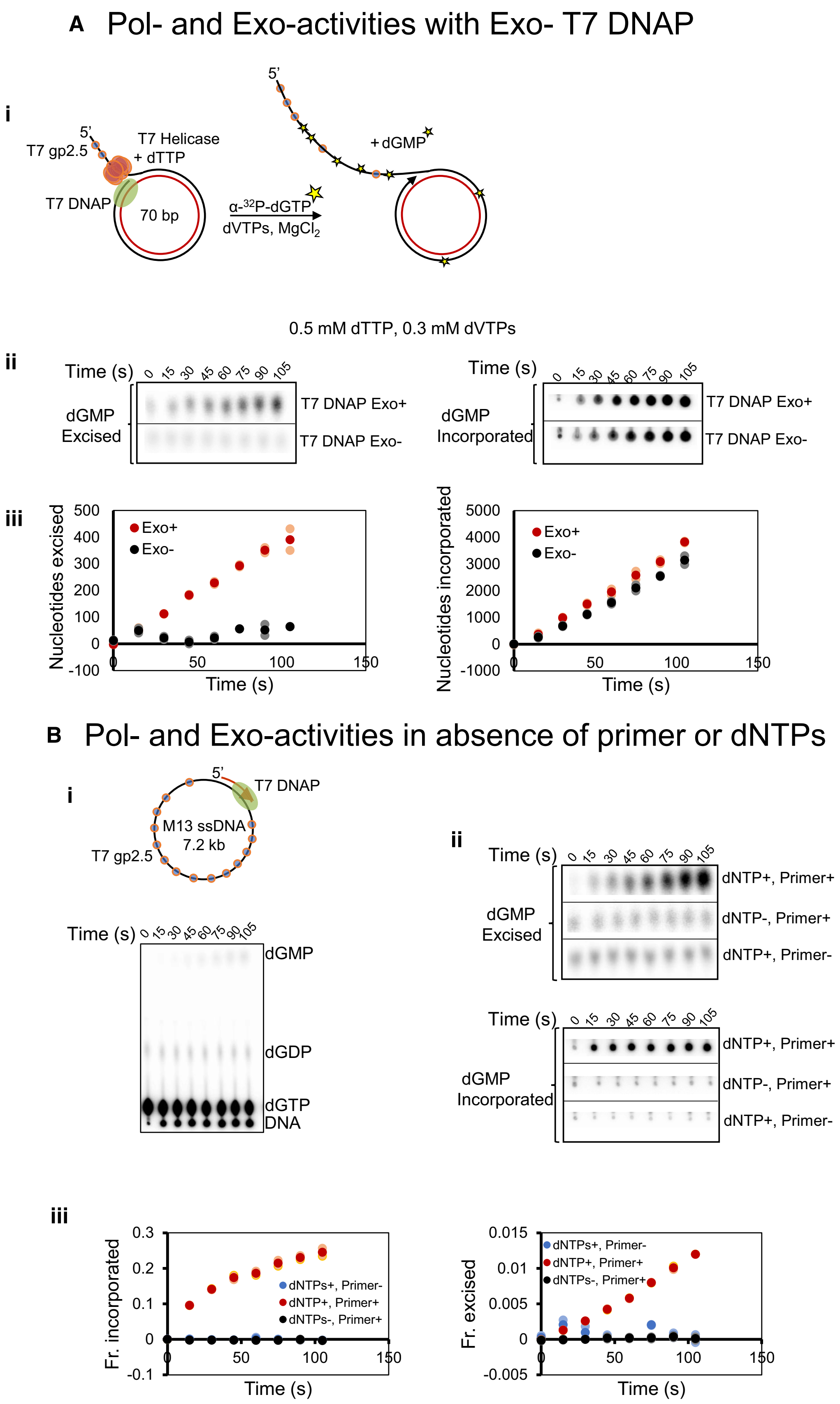

Figure EV2.

 © 2020 The Authors

Figure EV3. Polymerase and exonuclease activity assays in the presence of α-Thio dNTPs.

A Phosphorothioate linkage is resistant to 3′–5′ exonuclease activity of T7 DNAP. (i) and (ii) Design of the DNA substrates used for the experiments. A mismatched 35-mer/65-mer primer/template with the primer having a fluorescein moiety at 5′ end and a phosphorothioate linkage at 3′ end was used. Control primer/template did not have a phosphorothioate linkage. Excision reactions were conducted on a rapid quenched-flow apparatus and analyzed on 24% denaturing polyacrylamide gels. (iii) Relative intensities of 35-mer primer left at different time points.

B Real-time helicase unwinding traces (i) and determined unwinding rates with dTTP and dTTPαS (ii). Moles of nucleotides incorporated (iii) and excised (iv) per mole of substrate DNA during leading strand synthesis reactions performed in the presence of α-thio-dNTP (dNTPαS).

Data information: Bars show the data from two individual experiments. Circles in two shades of the same color show data points from two individual experiments. Mean data are fitted to linear trends.

© 2020 The Authors

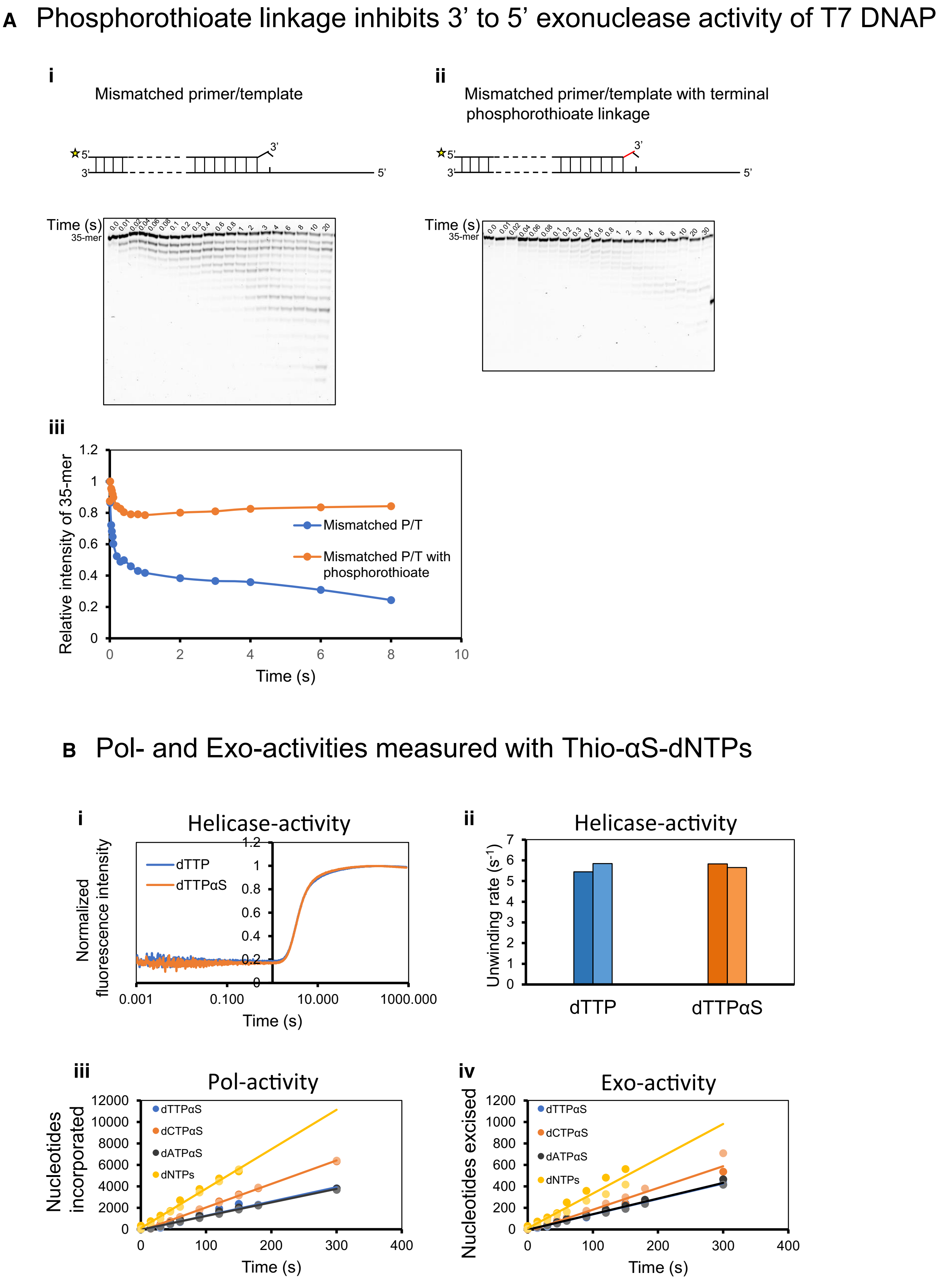

Figure EV3.

 © 2020 The Authors

Figure EV4. Primer-end transfer to Exo-site initiates from pre-translocated DNAP.

A Matched primer/template substrate used for determining rates of pyrophosphorolysis and excision. The asterisk at 5′ end of primer represents fluorescein moiety (i). The intensities of the bands quantitated from denaturing gels shown in Fig 4 were used to fit the data in the model shown below the DNA substrate. The data fit best to a model that assumed two populations, a predominant population catalyzing the reactions at a fast rate and another one at a slower rate. Hence, the rate constants from the faster population were used to correlate excision and pyrophosphorolysis in Fig 4E–G. Traces are showing the fitting of kinetic data from pyrophosphorolysis (ii) and excision (iii) reactions.

B Destabilization of pre-translocation state inhibits exonuclease activity. (i) Crystal structure of DNA bound T7 DNAP showing the interaction of the n+1 base of the template with DNAP residues. (ii) and (iii) Top, Primer–template design used in the experiment. 5′ end of the primer was fluorescein-labeled (represented with an asterisk). The abasic template had an abasic site at +1 position (iii, marked with red). Bottom, sequencing gel showing the pyrophosphorolysis of the 25-mer primer for control (ii, left) and abasic template (iii, left). Excision reactions performed with T7 DNAP exo+ in the absence of dNTPs, as seen on 24% polyacrylamide sequencing gels for reactions performed with control (ii, right) and abasic (iii, right) templates.

© 2020 The Authors

A Fitting of pre-steady state kinetic data from pyrophosphorolysis and exonucleolysis reactions

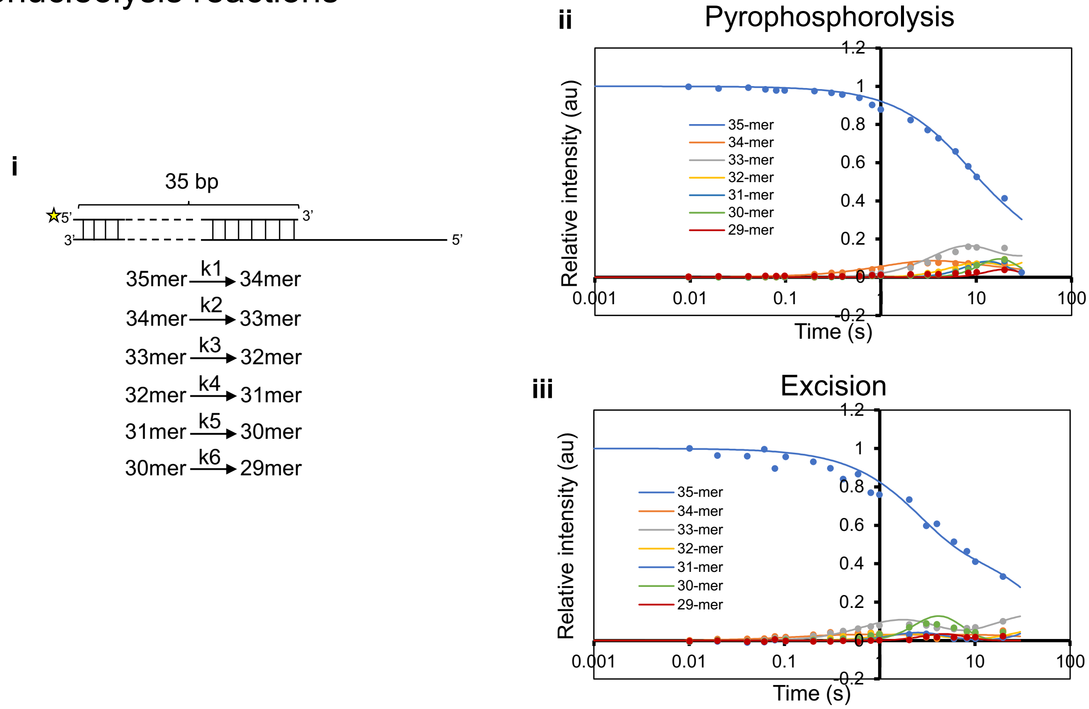

B Abasic site at *n+1* position in template inhibits pyrophosphorylation and excision

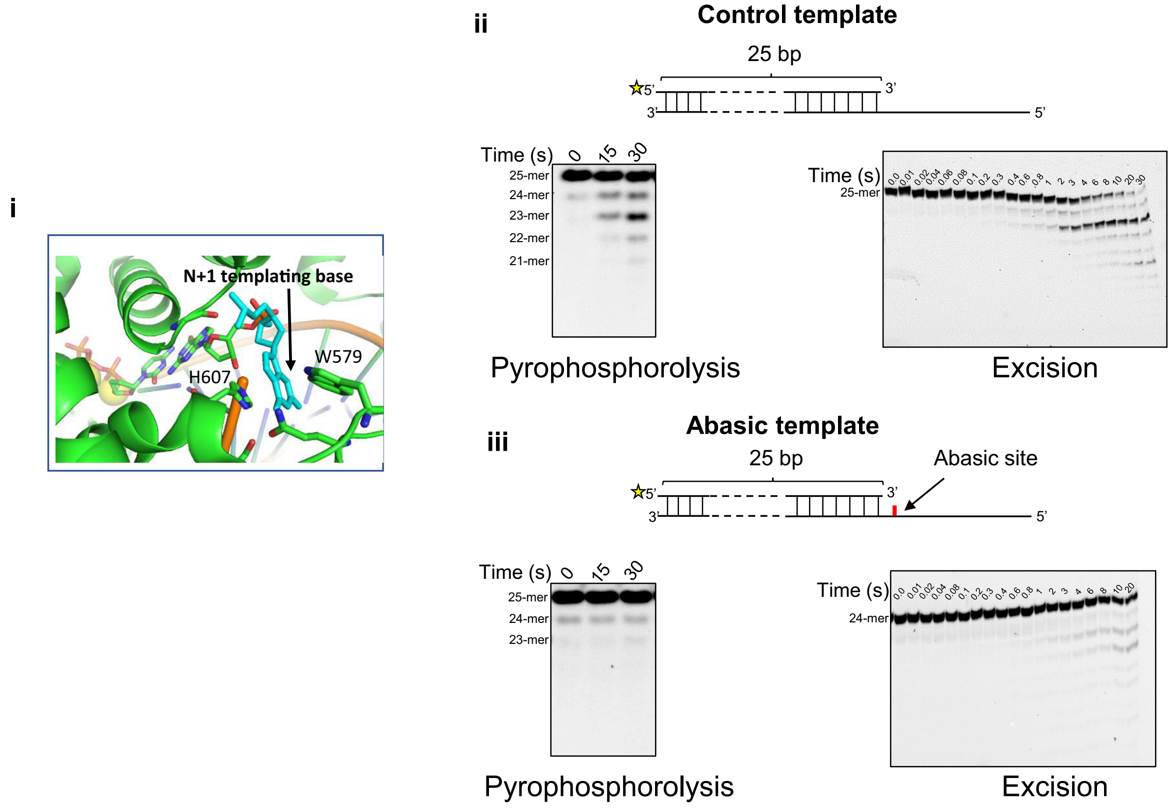

Figure EV4.

 © 2020 The Authors

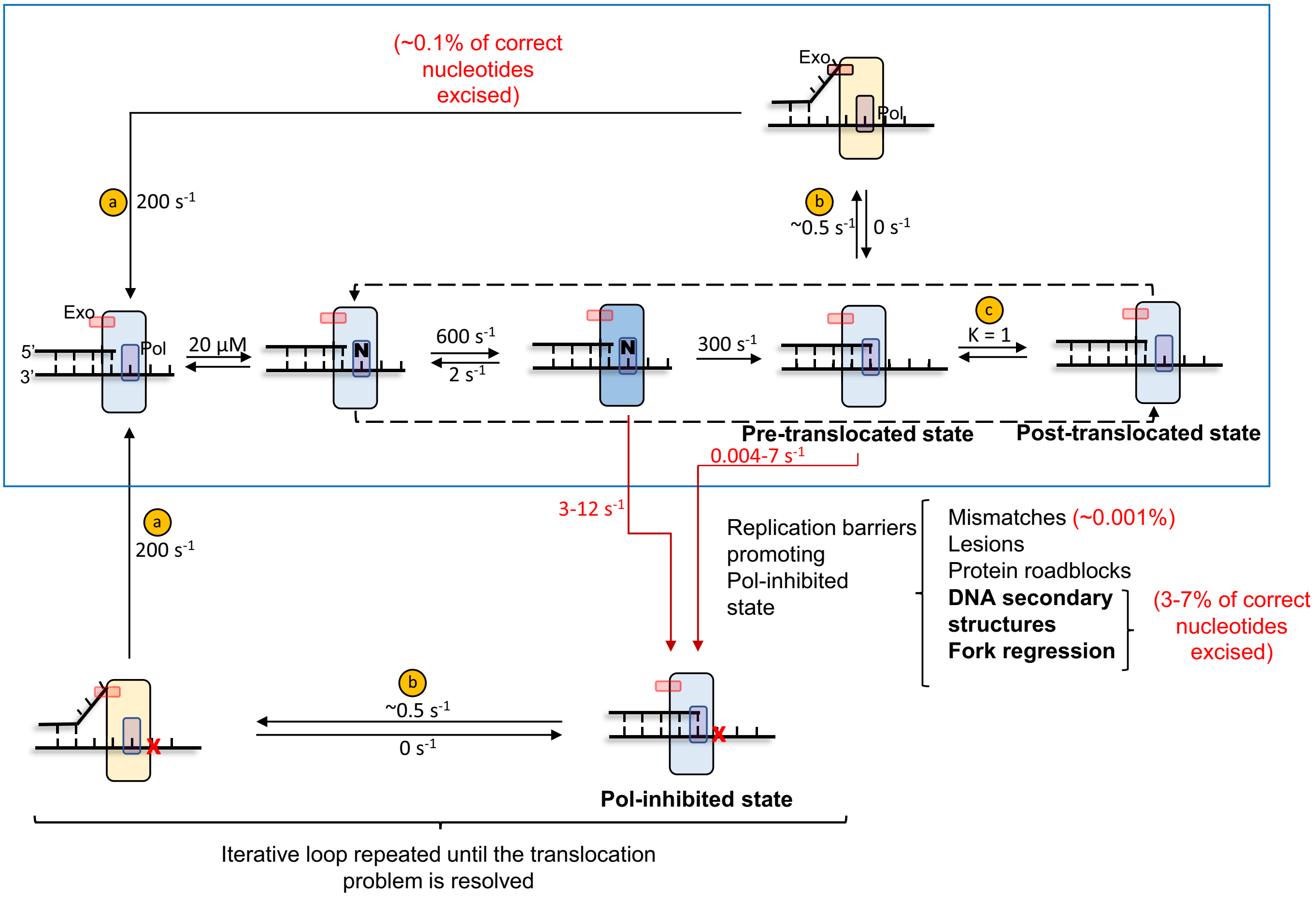

Figure EV5. Fitting of data to translocation-regulated model of active-site switching.

The model is showing the normal DNA synthesis pathway (in the blue selection) and the proposed alternative route via Pol-inhibited states. The values of kinetic parameters used for fitting data in the two models tested in the result section (Fig 5) are shown for each step. Values in steps “a” and “c” are taken from Donlin *et al* (1991) and Lieberman *et al* (2014), respectively. Rest of the values are from Johnson (2010). Step “b” is sequence-dependent and was arbitrarily put. The fittings were performed with the chi-square threshold limit of 0.67 set in FitSpace module of Kintek Explorer (Johnson *et al*, 2009a). The range of the floated parameters determined from the fitting of the data to model 2 (presented in Fig 5A) is in red font.

© 2020 The Authors
